# Development of a versatile tool for the simultaneous differential detection of *Pseudomonas savastanoi *pathovars by End Point and Real-Time PCR

**DOI:** 10.1186/1471-2180-10-156

**Published:** 2010-05-28

**Authors:** Stefania Tegli, Matteo Cerboneschi, Ilaria Marsili Libelli, Elena Santilli

**Affiliations:** 1 Dipartimento di Biotecnologie Agrarie, Sez. Patologia vegetale, Laboratorio di Patologia Vegetale Molecolare, Università degli Studi di Firenze, Via della Lastruccia 10, 50019 Sesto Fiorentino, Firenze, Italy

## Abstract

**Background:**

*Pseudomonas savastanoi *pv. *savastanoi *is the causal agent of olive knot disease. The strains isolated from oleander and ash belong to the pathovars *nerii *and *fraxini*, respectively. When artificially inoculated, pv. *savastanoi *causes disease also on ash, and pv. *nerii *attacks also olive and ash. Surprisingly nothing is known yet about their distribution in nature on these hosts and if spontaneous cross-infections occur. On the other hand sanitary certification programs for olive plants, also including *P. savastanoi*, were launched in many countries. The aim of this work was to develop several PCR-based tools for the rapid, simultaneous, differential and quantitative detection of these *P. savastanoi *pathovars, in multiplex and *in planta*.

**Results:**

Specific PCR primers and probes for the pathovars *savastanoi*, *nerii *and *fraxini *of *P. savastanoi *were designed to be used in End Point and Real-Time PCR, both with SYBR^® ^Green or TaqMan^® ^chemistries. The specificity of all these assays was 100%, as assessed by testing forty-four *P. savastanoi *strains, belonging to the three pathovars and having different geographical origins. For comparison strains from the pathovars *phaseolicola *and *glycinea *of *P. savastanoi *and bacterial epiphytes from *P. savastanoi *host plants were also assayed, and all of them tested always negative. The analytical detection limits were about 5 - 0.5 pg of pure genomic DNA and about 10^2 ^genome equivalents per reaction. Similar analytical thresholds were achieved in Multiplex Real-Time PCR experiments, even on artificially inoculated olive plants.

**Conclusions:**

Here for the first time a complex of PCR-based assays were developed for the simultaneous discrimination and detection of *P. savastanoi *pv. *savastanoi*, pv. *nerii *and pv. *fraxini*. These tests were shown to be highly reliable, pathovar-specific, sensitive, rapid and able to quantify these pathogens, both in multiplex reactions and *in vivo*. Compared with the other methods already available for *P. savastanoi*, the identification procedures here reported provide a versatile tool both for epidemiological and ecological studies on these pathovars, and for diagnostic procedures monitoring the asymptomatic presence of *P. savastanoi *on olive and oleander propagation materials.

## Background

Olive knot disease is a plant disease characterized by hyperplastic symptoms mainly on twigs, young branches and the trunk, more rarely on leaves and fruits, of olive trees (*Olea europaea *L.), which exists worldwide wherever this crop is cultivated. This disease is particularly damaging as far as quantitative and qualitative production is concerned [1,2], and causes heavy losses in the countries of the Mediterranean basin where olive plants are extensively cultivated. The causal agent is the plant pathogenic bacterium *Pseudomonas savastanoi *pv. *savastanoi *(*Psv*) [3,4], isolated and described for the first time by Luigi Savastano [5,6]. *Psv *enters and infects plants generally through wounds of different origin (*i.e*. pruning and mechanical wounds, frost injuries, leaf scars) [7]. The pathological process depends on the expression of bacterial *hrp *genes [8], and the development of the spherical knots is caused by phytohormones (3-indoleacetic acid and cytokinins) synthesized by *Psv*, that trigger uncontrolled proliferation of the cells surrounding the site of infection [9-13]. In the species *P. savastanoi *were also included isolates from oleander (*Nerium oleander *L.), ash (*Fraxinus excelsior *L.) and other plants, such as privet (*Ligustrum japonicum *Thunb.), *Jasminum *spp. and *Retama sphaerocarpa *(Boiss.) L., and the taxonomy and the classification of this bacterium have been controversial for a long time. The strains isolated from olive, oleander and ash can be differentiated according to a series of characteristics concerning host range, production of phytohormones and bacteriocins, assimilation of different carbon sources, monoclonal antibodies, analysis of whole cell fatty acids, DNA relatedness, low molecular weight restriction fragments, restriction fragment length polymorphism (RFLP) and fluorescent amplified fragment length polymorphism (f-AFLP) [3,12,14-24]. Moreover the genetic diversity of strains isolated from olive trees was recently deeply investigated [25-28]. According to all these data, the name *Psv *is now used to indicate isolates from olive, while the names *P. savastanoi *pv. *nerii *(*Psn*) and *P. savastanoi *pv. *fraxini *(*Psf*) are accepted for those strains isolated from oleander and ash, respectively [4].

The strategies to control olive knot mainly aim to reduce the spread of the disease, with general cultural practices such as pruning, particularly of affected branches, and the conventional use of copper compounds. Up to now no commercial olive cultivars resistant to *Psv *are available yet, but some researches on this topic have been reported [29-32]. Sources of inoculum for new infections are represented by *Psv *populations surviving within the young knots, but also by *Psv *naturally resident on healthy olive trees as epiphyte on the phylloplane, on the surfaces of stems and olive fruits. *Psv *epiphytic populations are important sources of inoculum for new infections, and their density is related to the season and the age of leaves, with the greatest damages observed when weather conditions were conducive both for the growth of *Psv *as epiphyte and its entry into the olive bark [33-38]. Thus, also considering the increasing spread of resistance to copper compounds among *P. syringae *pathovars and related bacteria [39,40], sensitive and specific methods to monitor *Psv *natural epiphytic population on olive trees are needed to contribute to the successful preventive control and management of this disease. Moreover, *Psv *is among the infective agents of olive, whose absence has to be ascertained for the production of certified olive plants [41].

Traditional microbiological methods for the detection and identification of *Psv *are available [42,43], but they have low sensitivity and specificity, and they are quite time consuming. For this reason some protocols were developed for the detection of *Psv*, by conventional, enriched and nested PCR, working also *in planta *and in asymptomatic tissues [44-46]. These assays showed high levels of sensitivity, but they were unsuitable to accurately and reliably quantify the target phytopathogen. Moreover all these assays, as well as a sensitive and quantitative Real-Time PCR procedure developed for *Psn *detection in oleander plants [47], used primers designed on the sequence of *iaaL *gene, which encodes the conversion of IAA to IAA-lysine. But being this target common to all the isolates of *Psv*, *Psn *and *Psf*, none of these methods results to be pathovar-specific, while it is known that under experimental conditions *Psn *strains are able to infect olive [24], and that *Psf *strains are able to multiply in olive bark when artificially inoculated, although to a lower level than strains isolated from olive or oleander [21]. Since nothing is known yet about their distribution in nature on the different host plants and whether spontaneous cross-infections occur, pathovar-specific molecular protocols for the discrimination of *Psv*, *Psn *and *Psf *would be urgently needed, firstly to clarify this aspect of their epidemiology and then to avoid false positive results derived from the application of the above mentioned detection methods in sanitary certification programmes.

In this paper we describe the development of reliable PCR-procedures for the specific discrimination and quantification of *Psv*, *Psn *and *Psf*, both *in vitro *and *in planta *as epiphytes, by End Point PCR and Real-Time PCR, using two different technologies, the SYBR^® ^Green I detection dye and three pathovar-specific TaqMan^® ^hybridisation probes. Primers and probes specific for *Psv, Psn *and *Psf *were designed upon the sequence data of cloned fragments, previously amplified in Repetitive-sequence-based PCR (Rep-PCR) experiments with strains belonging to the three pathovars of *P. savastanoi *examined in this study using Enterobacterial Repetitive Intragenic Consensus (ERIC) primers [48]. These procedures have high sensitivity, specificity, rapidity and represent valid and innovative diagnostic tools that can suit all phytopathological laboratories, according to their equipment and skills, in order to promote and encourage the use of molecular detection methods for *Psv *in the frame of the certification programs for olive propagation materials.

## Results

### Identification of *P. savastanoi *pathovar-specific sequences by ERIC-PCR and design of pathovar-specific primers

**Figure 1:**
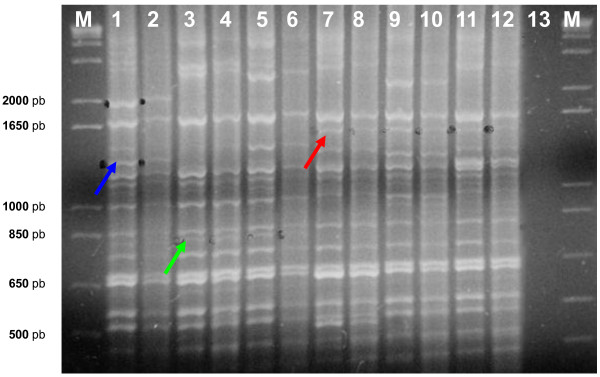
**ERIC-PCR fingerprintings of *P. savastanoi *strains belonging to the pathovars *Psv*, *Psn *and *Psf***. Pathovar-specific amplification bands are indicated by red, green and blue arrows for *Psv*, *Psn *and *Psf*, respectively. (See online for a colour version of this figure). M, marker 1 Kb Plus Ladder (Invitrogen Inc.). lanes 1-2: *Psf *strains; lanes 3-6: *Psn *strains; lanes 7-12: *Psv *strains; lane 13: DNA-free negative control.

The identities of *P. savastanoi *strains shown in Table 1 were confirmed by 16S rDNA sequencing and pathogenicity trials (data not shown). On these strains, Rep-PCR experiments with ERIC1R and ERIC2 primers were performed and the results referring to some representative strains for each *P. savastanoi *pathovar examined are shown in Figure 1. The genomic ERIC-PCR profiles were highly reproducible; they consisted of bands ranging in size from 400 to 5,000 bp and were pathovar-specific. For each *P. savastanoi *pathovar at least a single and unique band, appearing in all the strains belonging to the same pathovar, was detected. The sizes were approximately 1,600, 830 and 1,350 bp in *Psv*, *Psn *and *Psf*, respectively (Figure 1). These pathovar-specific bands were then separately isolated and purified from agarose gels, cloned and analyzed for their nucleotidic sequences composition. Each band was demonstrated to consist of several fragments of the same size but having different nucleotidic sequences, which were then individually DIG-labeled and used as probes in dot blot hybridization experiments performed under high stringency with the genomic DNAs of *Psv*, *Psn *and *Psf *previously blotted to nylon film (data not shown). The sequences assessed to be pathovar-specific in hybridization experiments were then analyzed using the BLASTN and BLASTX softwares, and further comparisons were made with the CLUSTALW2 program. According to the data so obtained and concerning their specificity, three ERIC-derived clones were selected, one for each pathovar [GenBank:FM253089; GenBank:FM253090; GenBank:FM253091]. Clone FM253090 from *Psn *did not show any significant homology with any nucleotidic or aminoacidic sequence present in the main databases. Clone FM253089 from *Psv *had a quite significant homology (82-67%) near its 3' end with putative transcriptional regulators belonging to the TetR family, while no homology was ever detected with any nucleotidic sequence. On the contrary, clone FM253091 from *Psf *showed a significant homology both in BLASTX and BLASTN analysis (88-74% and 99-51%, respectively) with sequences related to proteins belonging to the so called "VirD4/TraG family" of Type Four Secretion System [49]. By hybridization experiments clones FM253089 and FM253090 were demonstrated to be located on bacterial chromosome, while clone FM253091 was located on a plasmid of about 24 kb (data not shown). These three clones were further analysed in order to identify for each of them conserved regions specifically present in all the strains of the same pathovar, then used to design pathovar-specific primers and probes for End Point and Real-Time PCR (Table 2).

**Table 1:** Bacteria used in this study.

**Strain**^**a**^	Host plant of isolation	Geographical origin	End Point PCR			Real-Time PCR		
***P.savastanoi ***pv. ***savastanoi***			**pathovar- specific primer pairs**			**pathovar- specific primers/probes**		
			***Psv***	***Psn***	***Psf***	***Psv*-RT**	***Psn*-RT**	***Psf*-RT**
			
ITM317, IPVCT-3, LPVM22, LPVM510, LPVM602, ES47^b^, ES49^b^, ESB50^b^, PvBa223	olive	Southern Italy	+	-	-	+	-	-
Legri1^b^, Legri2^b^, MC1^b^, MC33^b^, MC72^b^, MC80^b^, LPVM15, LPVM20	olive	Central Italy	+	-	-	+	-	-
								
ITMKS1, ITMKL1, ST2^ b^	olive	Greece	+	-	-	+	-	-
1657-8^ c^	olive	Spain	+	-	-	+	-	-
DAR7635^d^	olive	Australia	+	-	-	+	-	-

*P. savastanoi *pv. *nerii*								
								
ITM519, IPVCT-99, ESC8^b^, ESC6^b^, ESC43^b^, ESB60^b^, LPVM12, LPVM33, LPVM71, LPVM201, PvBa219	oleander	Southern Italy	-	+	-	-	+	-
ITM601, ES23^b^, LPVM103	oleander	Northern Italy	-	+	-	-	+	-
NCPPB640	oleander	Ex-Yugoslavia	-	+	-	-	+	-

*P. savastanoi *pv. *fraxini*								
								
NCPPB1006, NCPPB1464	ash	United Kingdom	-	-	+	-	-	+
PD120	ash	The Netherlands	-	-	+	-	-	+
CFBP1838, CFBP2093	ash	France	-	-	+	-	-	+
MCa3^b^, MCa4^b^	ash	Italy	-	-	+	-	-	+

*P. savastanoi *pv. *phaseolicola *1449B^e^	*Lablab purpureus*	Ethiopia	-	-	-	-	-	-
*P. savastanoi *pv. *glycinea *PG4180^f^	soybean	New Zealand	-	-	-	-	-	-
Unidentified bacteria^g^	olive, oleander, ash	Central Italy	-	-	-	-	-	-

^a ^CFBP, Collection Française de Bactéries Phytopathogènes, INRA, Angers, France; IPVCT, Culture collection of Dipartimento di Scienze e Tecnologie Fitosanitarie, Università degli Studi di Catania, Italy (from V. Catara); ITM, Culture collection of Istituto Tossine e Micotossine da Parassiti vegetali, C. N. R., Bari, Italy (from A. Sisto); LPVM, Culture Collection of Laboratorio di Patologia Vegetale Molecolare, Dipartimento di Biotecnologie Agrarie, Università degli Studi di Firenze; NCPPB, National Collection of Plant Pathogenic Bacteria, York, UK http://www.ncppb.com/; PD, Culture collection of Plant Protection Service, Wageningen, The Netherlands; PVBa, Culture Collection of Dipartimento di Patologia Vegetale, Università degli Studi di Bari, Italy (from A. Sisto).

^b ^from E. Santilli and M. Cerboneschi

^c ^from M. M. Lopez

^d ^from E. J. Cother

^e ^from R. W. Jackson

^f ^from M. S. Ullrich

^g ^bacterial epiphytes naturally occurring *P. savastanoi *host plants and isolated as described in Methods.

**Table 2:** Nucleotide sequences of PCR primers and probes used and developed in this study.

Primer/Probe^a^	Sequence (5'-3')	Position^b^	Product size (bp)	Accession Number
*Psv*F	GGCGATGTTCTCAGCGGATTTG	24	388	FM253081
*Psv*R	GATCAAGTGTCCAAGGAAGTGAAGG			FM253082
*Psv*RT-F	CGGATTTGGTTTGCGGGGTA	38	298	FM253083
*Psv*RT-R	AATGGGGTGACACTAAAAATTGTGAA			FM253084
*Psv*RT-P	(HEX)CTCGTGCGATCTAAACAGCCGTAGC(BHQ-1)^c^	278		FM253085
				
*Psn*F	ACCCCTCATTGTAACGGATG	1	349	AM051225
*Psn*R	TCCCCGGAATTCAACACTTA			AM051226
*Psn*RT-F	GCTCATTCGCTTGTTATCACTTCA	181	169	AM086621
*Psn*RT-R	TCCCCGGAATTCAACACTTA			AM051226
*Psn*RT-P	(FAM)TACGCCCGACGCCCGAGCCA(BHQ-1)^c^	206		FM253086
				
*Psf*F	CGCCTGCTGTACTCCTCGG	1	412	AM055834
*Psf*R	TCGACCTGTCTAAGGCCC			AM055835
*Psf*RT-F	CAGCTCATCCATTAATAGGGCAAG	207	227	AM086622
*Psf*RT-R	GGGCAGTGTCAGGGGATG			FM253088
*Psf*RT-P	(Texas Red)CTTGTACCGAAGCGTGCCGTCTGC(BHQ-2)^c^	237		FM253087

^a ^F, forward; R, reverse; RT, RealTime; P, probe.

^b ^Starting nucleotide position of forward primers and TaqMan^®^ probes on target sequences.

^c ^BHQ-1 and BHQ-2 are quencher molecules available from the manufacturer.

### End Point PCR assays for *Psv*, *Psn *and *Psf *specific detection

**Figure 2:**
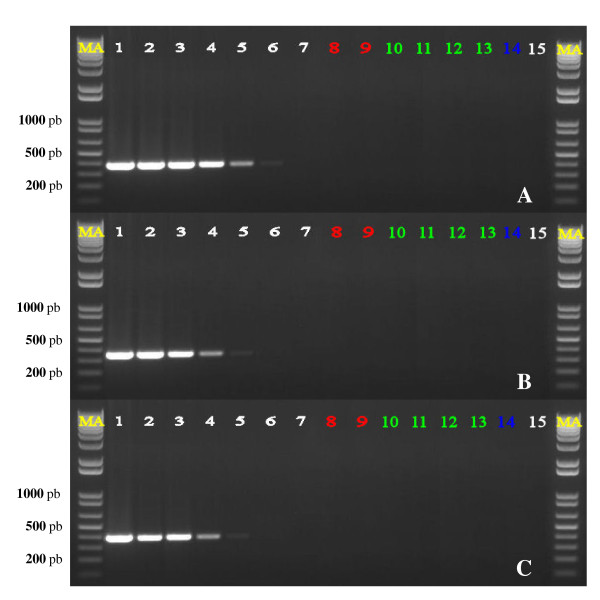
**Specificity and detection limit of End Point PCR assays**.(A) primer set *Psv*F/*Psv*R on strain *Psv *ITM317; (B) primer set *Psn*F/*Psn*R on strain *Psn *ITM519; (C) primer set *Psf*F/*Psf*R on strain *Psf *NCPPB1464. M, marker 1 Kb Plus Ladder (Invitrogen Inc.). lanes 1-7: genomic DNA from the target *P. savastanoi *pathovar (serial tenfold dilutions, from 50 ng to 0.05 pg per reaction); lanes 8-9: genomic DNA from the non-target *P. savastanoi *pathovars (50 ng/reaction); lanes 10-13: plant genomic DNA (50 ng/reaction), from olive, oleander, ash and oak, respectively; lane 14: genomic DNA (50 ng/reaction) from a pool of bacterial epiphytes isolated in this study from olive (A), oleander (B) and ash leaves (C); lane 15, DNA-free negative control;

In order to obtain information about their specificity and sensitivity, the primer pairs *Psv*F/*Psv*R, *Psn*F/*Psn*R and *Psf*F/*Psf*R, whose sequences and descriptions are reported in Table 2, were evaluated in End Point PCR assays using as template the genomic DNA of strains *Psv *ITM317, *Psn *ITM519 and *Psf *NCPPB1464, which are representative of their pathovars. For each primer set several serial tenfold dilutions of genomic DNA (from 50 ng to 0.05 pg) of the isolate belonging to the pathovar for which that primer pair was supposed to be specific were used as template. Genomic DNAs (50 ng/reaction) extracted from each one of the other two *P. savastanoi *isolates, from olive, oleander, ash and oak, and from pooled samples of bacterial epiphytes isolated from these plants were also tested. The results obtained are shown in Figure 2, where 1/5 of the reaction volume for each sample tested was loaded onto the agarose gel, to better compare the data. A single amplicon was produced with each primer pair of the three tested, specifically when the DNA template was from the *P. savastanoi *pathovar for which the primer set was designed. The size of each amplicon was as expected: 388 bp for *Psv*F/*Psv*R, 349 bp for *Psn*F/*Psn*R and 412 bp for *Psf*F/*Psf*R, with DNA template from strains *Psv *ITM317, *Psn *ITM519 and *Psf *NCPPB1464, respectively. No amplicons were ever obtained with no target DNA, either from olive, oleander, ash and oak or from the pools of bacterial epiphytes from *P. savastanoi *host plants. The sensitivity of these PCR assays was estimated by determining the lowest amount of DNA template detected, that was found to be approximately 5 pg for the primer sets *Psn*F/*Psn*R and *Psf*F/*Psf*R, and 0.5 pg for the pair *Psv*F/*Psv*R, here corresponding to DNA concentrations of 0.2 and 0.02 pg/μl, respectively (Figure 2).

For further testing the pathovar-specificity of the End Point PCR detection methods developed in this study, genomic DNAs from the bacteria listed in Table 1 were also assayed (50 ng/reaction). Forty-four *P. savastanoi *strains, belonging to three *P. savastanoi *pathovars here examined and having different geographic origins, were tested. For comparison, strains 1449B of *P. savastanoi *pv. *phaseolicola *(*Psp*) and PG4180 *P. savastanoi *pv. *glycinea *(*Psg*), taxonomically closely related to the pathovars of our interest, were also included in this study. In Table 1 the results obtained are schematically reported: the signs + and - indicate the presence or absence of the expected amplicons, respectively. The pathovar-specificity of each primer pair was confirmed and all the strains belonging to a pathovar were correctly identified when tested with the primer set supposed to be specific for that pathovar. No unspecific amplifications were ever generated, confirming that these End Point PCR assays are highly specific and able to discriminate strains belonging to *Psv*, *Psn *and *Psf*.

### SYBR^® ^Green Real-Time PCR assays for *Psv*, *Psn *and *Psf *specific detection

**Figure 3:**
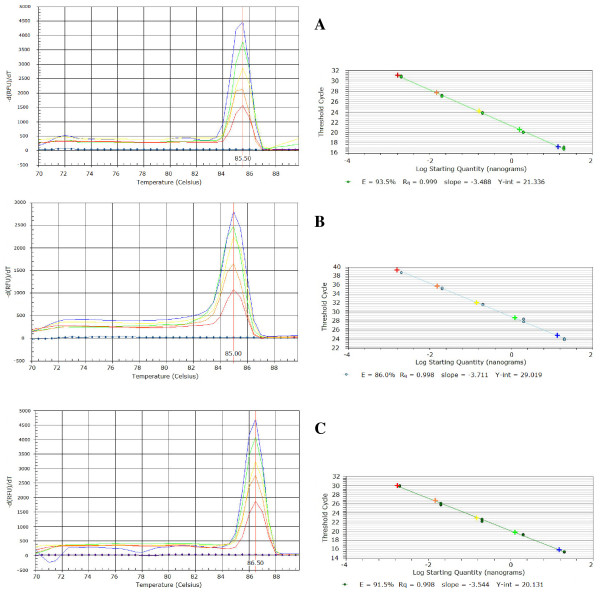
**Melting temperature analysis and quantitative standard curves of SYBR^® ^Green Real-Time PCR assays**.(A) primer set *Psv*RT-F/*Psv*RT-R on strain *Psv *ITM317; (B) primer set *Psn*RT-F/*Psn*RT-R on strain *Psn *ITM519; (C) primer set *Psf*RT-F/*Psf*RT-R on strain *Psf *NCPPB1464. Quantitative thermal dissociation curves were represented plotting fluorescence derivative values [-d (fluorescence units)/d (time)] *versus *temperature, obtained with DNA from the target *P. savastanoi *pathovar, extracted by thermal lysis from 10^3 ^to 10^7 ^CFU per reaction (red, orange, yellow, green and blue lines, respectively) and with no target DNAs (blue diamond), extracted from the two other *P. savastanoi *pathovars, from olive (A), oleander (B) and ash (C) and from a pool of bacterial unidentified epiphytes isolated from the same plants (from olive, oleander and ash in A, B and C, respectively). Standard curves were generated by plotting the Ct values *versus *the log of genomic DNA concentration of each tenfold dilution series in the range of linearity (from 50 ng to 5 pg per reaction). The Ct data obtained with target DNA from 10^3 ^to 10^7 ^CFU per reaction were reported (**+**). (See online for a colour version of this figure).

On the ERIC pathovar-specific sequences and internal to the annealing sites for the End Point PCR primers, three primer sets to be used in Real-Time PCR were designed, one for each *P. savastanoi *pathovar examined. The specificity of these primer pairs, named *Psv*RT-F/*Psv*RT-R, *Psn*RT-F/*Psn*RT-R, *Psf*RT-F/*Psf*RT-R (Table 2), was preliminarily assessed by BLAST analysis. Then these primer sets were tested in Real-Time PCR runs with SYBR^® ^Green as fluorescent marker and 1 μl of DNA template extracted from 1 ml of titrated suspensions (corresponding to about 10^3 ^to 10^7 ^CFU/reaction) of strains *Psv *ITM317, *Psn *ITM519 and *Psf *NCPPB1464. Since SYBR^® ^Green binds to the minor grooves of a DNA double-chain as it is forming, this fluorescent dye can bind to all amplicons produced in a PCR reaction. Therefore, the specificity of detection can be provided by a pair of primers only when the increase in fluorescence is generated by a single amplicon with a distinct melting temperature (Tm). For this reason dissociation analysis is crucial in SYBR^® ^Green PCR experiments. The melting curves obtained with the primer pairs developed in this study are shown in Figure 3.

For all the five different cell concentrations a single melting peak at 85.5°C (± 0.1) was observed with the primer pair *Psv*RT-F/*Psv*RT-R and DNA extracted from isolate *Psv *ITM317, to indicate that the total fluorescent signal was contributed by specific amplicons. No signals were recorded in melting point analysis with the set *Psv*RT-F/*Psv*RT-R in DNA-free control and when no target DNAs were used as template (Figure 3). The pair *Psn*RT-F/*Psn*RT-R obtained a similar specificity, giving a unique melting peak at 85.0°C (± 0.1) only with DNA from strain *Psn *ITM519, as well as the primer set *Psf*RT-F/*Psf*RT-R that originated a single peak at 86.5°C (± 0.1) only with DNA from strain *Psf *NCPPB1464. In these cases as well, DNA-free controls and the DNAs supposed not to be specific for these primers tested negative (Figure 3), unless spiked with target DNA specific for the primer pair used (data not shown). The pathovar-specificity of each primer pair was further confirmed using as template DNAs from the bacteria listed in Table 1. The results obtained are schematically reported for each strain; the signs + and - indicate the presence or absence of the expected melting peak, respectively (Table 1). Moreover the amplicons produced by SYBR^® ^Green Real-Time PCR were visualized by gel electrophoresis. Single bands of the expected sizes of 298, 169 and 227 bp were specifically generated with the primer sets *Psv*RT-F/*Psv*RT-R, *Psn*RT-F/*Psn*RT-R, *Psf*RT-F/*Psf*RT-R and isolates belonging to *Psv*, *Psn *and *Psf*, respectively, and no aspecific amplification products were ever observed (data not shown).

In Figure 3 the sensitivity of each pathovar-specific primer pair is also represented. For each primer set increasing amounts of the specific target DNA corresponded to higher melting peaks having the same Tm, and DNA as small as that extracted from 10^3 ^CFU could be easily detected.

The standard curves for the absolute quantification of the DNA target by SYBR^® ^Green Real-Time PCR detection methods here developed were generated by evaluating the Ct values *versus *the log of DNA concentration of each tenfold dilution series (from 50 ng to 5 fg per reaction). As shown in Figure 3 the linearity of the quantification was demonstrated over a range of five logs (from 50 ng to 5 pg/reaction), with excellent correlation coefficients (R^2^) of 0.999, 0.998 and 0.998 for pathovar-specific primer sets *Psv*RT, *Psn*RT and *Psf*RT, respectively. The slopes of the standard curves (between -3.488 and -3.711) were equivalent to PCR efficiencies ranging from 93.5 to 86.0%, to indicate that these SYBR^® ^Green Real-Time PCR assays are solid even with low DNA target concentrations, as further confirmed when the Ct values obtained with DNA from titrated suspensions were reported on the plots (Figure 3).

### TaqMan^® ^Real-Time PCR assays for *Psv, Psn *and *Psf *specific detection

**Figure 4:**
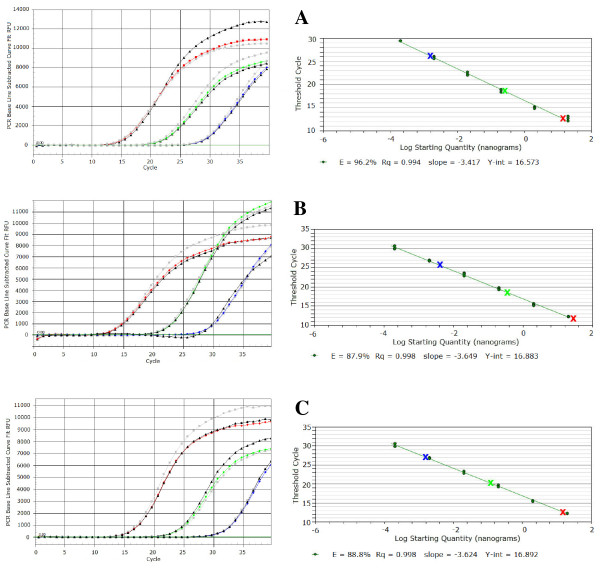
**Sensitivity of TaqMan^® ^probes *Psv*RT-P (A), *Psn*RT-P (B) and *Psf*RT-P (C)**. Sensitivity was assessed by using DNA extracted from strains *Psv *ITM317 (A), *Psn *ITM519 (B) and *Psf *NCPPB1464 (C). Amplification curves of DNA from target *P. savastanoi *pathovar extracted from 10^3^, 10^5 ^and 10^7 ^CFU per reaction and used pure (red diamond, green diamond and blue diamond, respectively) or spiked with no-target *P. savastanoi *pathovars DNA (50 ng/reaction each) (black diamond) or with DNA from the host plant of target *P. savastanoi *pathovar and from a pool of bacterial epiphytes present on this plant (50 ng/reaction each) (grey square). (See online for a colour version of this figure). Standard curves were generated by plotting the Ct values *versus *the log of genomic DNA concentration of each tenfold dilution series in the range of linearity (from 50 ng to 0.5 pg per reaction). The Ct data obtained with target DNA from 10^3 ^to 10^7 ^CFU per reaction were reported (X). (See online for a colour version of this figure).

SYBR^® ^Green Real-Time PCR is a reliable quantitative dye detection procedure, but unsuitable for multiple targets. In this perspective, on the sequences of the amplicons produced with the primer pairs *Psv*RT-F/*Psv*RT-R, *Psn*RT-F/*Psn*RT-R and *Psf*RT-F/*Psf*RT-R, the TaqMan^® ^probes *Psv*RT-P, *Psn*RT-P and *Psf*RT-P were designed to specifically identify *Psv*, *Psn *and *Psf *strains, respectively (Table 2). These fluorogenic probes were used in Real-Time PCR runs with 1 μl of DNA template, extracted from 1 ml of various titrated suspensions (corresponding to 10^3^, 10^5 ^and 10^7 ^CFU/reaction) of strains *Psv *ITM317, *Psn *ITM519 and *Psf *NCPPB1464. As shown in Figure 4, all these TaqMan^® ^probes provided the desired level of specificity, and Ct values ranging from 12 to 27 were generated with target DNA extracted from 10^3 ^to 10^7 ^CFU. No significant changes in Ct were ever observed when target DNA was spiked with DNA from no-target *P. savastanoi *pathovars (50 ng/reaction each) or with DNA from the host plant of the target *P. savastanoi *pathovar and from a pool of bacterial epiphytes present on this plant (50 ng/reaction each). Fluorescence always remained below the threshold values in DNA-free controls. The specificity was further confirmed using as template DNA (50 ng) extracted from the bacteria listed in Table 1: an increase in fluorescence, at the expected wavelength, was always obtained for all the strains of a *P. savastanoi *pathovar when the reaction mixture contained the TaqMan^®^ probe supposed to be specific for that pathovar, as schematically reported in Table 1. Negative results were always recorded using no-target DNAs.

The detection limits of TaqMan^® ^Real-Time PCR reactions were evaluated using different DNA amounts (from 50 ng to 5 fg per reaction) and standard curves for quantitative analyses were constructed for the three target *P. savastanoi *pathovars, using Ct values from three independent runs of PCR assays with three replicates each, plotted *versus *the log of DNA concentration of each tenfold dilution series. Standard curves showed a linear correlation between input DNA and Ct values over a range of six logs (from 50 ng to 0.5 pg per reaction), for all the pathovar-specific *P. savastanoi *TaqMan^® ^probes (Figure 4). Detection limits were always 500 fg of target DNA for *Psv*, *Psn*, and *Psf*, using the specific TaqMan^® ^probe, corresponding to about 10^2 ^bacterial genomes. Concerning R^2 ^values, these were 0.994, 0.998 and 0.998, with corresponding amplification efficiencies of 96.2%, 87.9% and 88.8%, for the probes *Psv*RT-P, *Psn*RT-P and *Psf*RT-P, respectively (Figure 4).

### Multiplex Real-Time PCR assays for *Psv*, *Psn *and *Psf *specific detection on artificially inoculated olive plants

**Figure 5:**
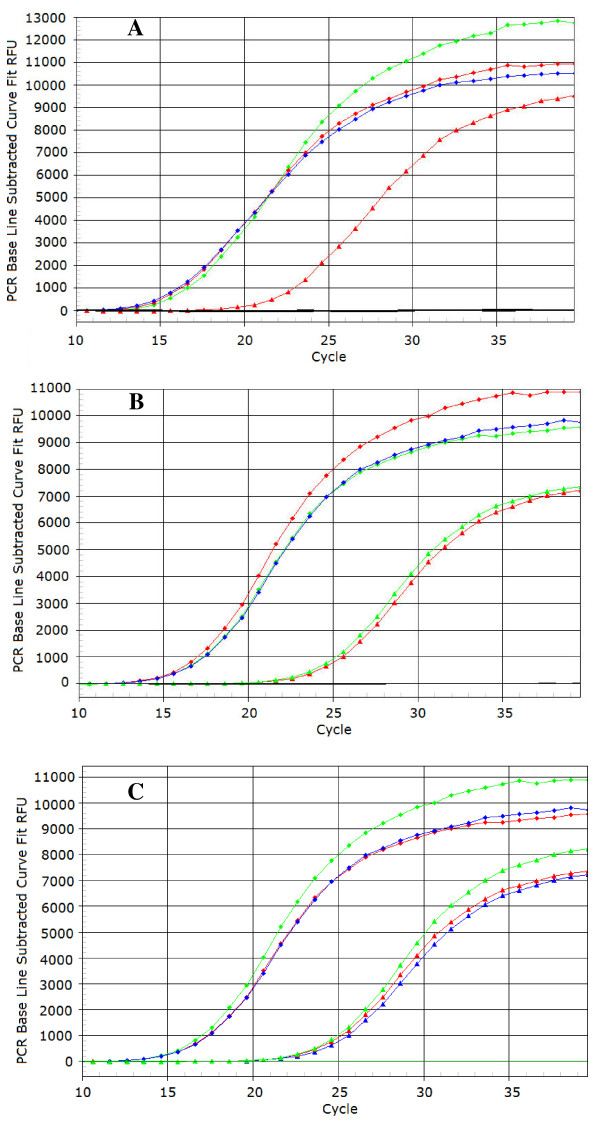
**Sensitivity of TaqMan^® ^probes in Multiplex Real-Time PCR assays**. Sensitivity of the TaqMan^® ^probes *Psv*RT-P, *Psn*RT-P and *Psf*RT-P was evaluated using *P. savastanoi *DNA extracted from olive leaves artificially inoculated with bacterial suspensions (10^7 ^CFU/leaf/strain) of *Psv *ITM317 (red triangle), *Psn *ITM519 (green triangle) and *Psf *NCPPB1464 (blue triangle), according to the following scheme. (A) *Psv *ITM317; (B) *Psv *ITM317 + *Psn *ITM519; (C) *Psv *ITM317 + *Psn *ITM519 + *Psf *NCPPB1464. Amplification curves obtained with DNA from *Psv *ITM317 (red diamond), *Psn *ITM519 (green diamond) and *Psf *NCPPB1464 (blue diamond) (50 ng/reaction each) and from water and uninoculated leaves (-) were also shown for comparison. (See online for a colour version).

In order to test whether the TaqMan^® ^probes here developed were compatible in multiplex reactions and whether they are applicable *in vivo*, some preliminary experiments were performed using as template DNA extracted by thermal lysis from olive leaves artificially inoculated with a bacterial suspension of strain *Psv *ITM317, alone or in combination with strains *Psn *ITM519 and *Psf *NCPPB1464, as described subsequently in Methods. The results obtained are reported in Figure 5, where all the three probes maintained the expected level of specificity in multiplex reactions as well, enabling the simultaneous detection of all the three target *P. savastanoi *pathovars, if present. The probe *Psv*RT-P gave always positive fluorescence signals at the expected wavelength, with almost the same Ct values in all the samples tested (Figure 5). The wavelength-specific fluorescence increase for the other two TaqMan^® ^probes, *Psn*-RT-P and *Psf*-RT-P, was observed only when the DNA template was extracted from olive leaves also inoculated with the *P. savastanoi *pathovars for which these probes were previously demonstrated to be specific (Figure 5). No differences were observed among the Cts obtained with the probe *Psv*RT-P and using as template the DNA extracted from the washings of leaves inoculated with strain *Psv *ITM317 alone or in combination with strains *Psn *ITM519 and *Psf *NCPPB1464 (Figure 5). For each probe, fluorescence always remained below the threshold values for the water controls, and for the DNA extracted from leaves inoculated with sterile water or uninoculated. Moreover the sensitivity of each TaqMan^® ^probe was unaffected by multiplexing, as assessed comparing the Ct values of the relative standard curves with those here obtained (Figure 4), both using pure DNA from *Pss *ITM317, *Psn *ITM519 and *Psf *NCPPB1464 (50 ng/reaction each), and DNA from the same pathovars extracted from olive leaves washings (corresponding to about 10^5 ^CFU per reaction for each *P. savastanoi *pathovar).

## Discussion

PCR-based methods are being increasingly used for detecting phytopathogenic bacteria, as recently reviewed by Palacio-Bielsa *et al*. [50]. Traditional methods are mainly based on the isolation of bacterial plant pathogens on semi-selective media, followed by morphological identification. Such methods are time consuming, usually require deep taxonomic expertise and are not able to give accurate results for pathogen quantification. On the contrary PCR-based protocols allow rapid, precise and direct bacterial detection, even *in planta*. Moreover, some of these techniques give quantitative results and are able to simultaneously detect up to five different target pathogens. As far as *P. savastanoi *is concerned, sanitary certification programs for olive and oleander mother plants and propagation materials already started in many countries [41,51,52], but the presence of *Psv *and *Psn *on these plants is still assessed mainly by visual inspection, looking for the typical hyperplastic knots. On the other hand, it was clearly demonstrated that the spread of the disease can also occur with asymptomatic propagation materials, where these bacteria can be found either as endophytes or as epiphytes [37,38,53-55]. Hence innovative detection protocols for *P. savastanoi *pathovars, which have very low detection limits and are able to obtain good results *in vivo*, are strongly needed and can be achieved only by PCR-based methods. All the PCR-based protocols up to now available for *P. savastanoi *are unable to differentiate *Psv*, *Psn *and *Psf *strains [44-47] and this could be an enormous limit for their routine applicability. In fact, while nothing is known yet about the natural distribution of *Psv*, *Psn *and *Psf *on the different hosts, cross-infections have been reported to occur under experimental conditions [21,24]. Thus, the availability of highly reliable pathovar-specific identification tests is both fundamental to definitely assess the natural host range of the *P. savastanoi *pathovars here examined and mandatory in light of the application of sanitary certification programs for olive and oleander.

In our study, a global approach was undertaken and for the first time a complex of PCR assays was developed for the highly specific and sensitive identification, detection and discrimination of the three pathovars *Psv*, *Psn *and *Psf*, in multiplex and quantitative reactions as well. These protocols were thought to be suitable both for research and for diagnostic purposes, with different laboratories applying End Point PCR or Real-Time PCR, using SYBR^® ^Green or pathovar-specific TaqMan^® ^probes, according to the aims of the work and to the available instrumentations and skills.

All these assays had a specificity score of 100%, since the only positive strains are those belonging to the *P. savastanoi *pathovar for which the PCR-based protocol was designed. To this purpose forty-four *P. savastanoi *strains were tested, having different geographical origins and belonging to the pathovars *Psv*, *Psn *and *Psf*. Negative results were always obtained with closely taxonomically related bacteria, such as *Psp *and *Psg*, and with bacterial epiphytes naturally occurring on leaves of olive, oleander and ash, as well as with DNA extracted from these plants and from oak, unless spiked with the *P. savastanoi *target DNA.

Concerning detection limits, positive results were obtained in End Point and Real-Time PCR with DNA amounts as small as 5 or 0.5 pg per reaction. Amounts of DNA corresponding to about 10^2 ^- 10^3 ^genomes per reaction could be easily detected in Real-Time PCR runs, with Ct values ranging from 30 to 39 and from 26 to 27, with SYBR^® ^Green or TaqMan^® ^probes, respectively, and according to the pathovar examined. The PCR protocols already available for the detection of *Psv *appeared to be slightly more sensitive than the assays developed in this study (from 10 to 10^2 ^CFU/ml on enriched samples of amended plant extracts), but it is to point that no reliable comparison was possible among data obtained in conventional PCR and Real-Time PCR [44-46]. Concerning the quantitative assay previously developed for *Psn*, based on the real time monitoring of the reaction and on the use of a TaqMan^® ^probe [47], it had higher performances (10 CFU/ml) than those obtained with the same technical approach in the present study, but after a one-day enrichment step. Since the primers and the probes here designed are extremely pathovar-specific, the TaqMan^® ^Real-Time procedures developed were also demonstrated to be an excellent quantitative method even when applied to multiplex assays for *Psv*, *Psn *and *Psf *specific detection and discrimination on artificially inoculated olive plants.

As a precaution against the possibility of false negative results, due to the presence of PCR inhibitors in the samples or to malfunctions of the thermal cycler, it is necessary not only to choose a DNA extraction procedure which would be able to eliminate PCR inhibitors, but also to ascertain their absence through a systematic monitoring. To this aim an internal amplification control (IAC) is sometimes included in the assay to test both PCR performance and inhibition [56]. But in some cases IAC was demonstrated to alter the precision and accuracy of the PCR assay itself, particularly in Real-Time PCR experiments [57]. For this reason in this study the possibility of false-negative results on positive samples was completely excluded using a different and more efficient strategy. Prior to be used in the detection assays, DNAs extracted from bacteria were tested by amplifying 16S rDNA with universal primers [58] and all the plant samples were tested as spiked with 50 ng/reaction of target *P. savastanoi *bacterial DNA: only those giving positive results were then further processed, while those testing negative were rejected and their DNA extraction repeated.

## Conclusions

The main novelty of this paper consists in the development of a versatile complex of PCR tools that for the first time will enable to easily and unequivocally distinguish *Psv*, *Psn *and *Psf *strains. The present End Point PCR assays are robust and suitable for routine culture confirmation purposes of these strains, avoiding laborious pathogenicity trials. Concerning the Real-Time PCR procedures, their high analytical sensitivity is definitely high enough for direct testing of plant materials, to detect the presence of these bacteria as epiphytes. Unlike the other assays published so far on *in vivo *PCR detection of *Psv *and *Psn*, the procedures here developed do not absolutely need any enrichment and nested-PCR, so that to avoid any possible contamination. Further experiments will focus on the upgrade of these protocols for the *in planta *detection of these bacteria as endophytes, encouraged by the results here obtained with the pathovar-specific TaqMan^® ^probes. Moreover because of their multiplexing activity, these probes are already available to yield new important insights into the epidemiology of *Psv*, *Psn *and *Psf *and of the diseases they caused.

## Methods

### Bacterial strains and pathogenicity tests

*P. savastanoi *strains used in this study are listed in Table 1. *P. savastanoi *strains were routinely grown on King's B agar (KB) [59], incubated at 26°C for 48 h. For liquid culture, bacteria were grown overnight on KB at 26°C on a rotary shaker (160 rpm). Bacterial suspensions were prepared from liquid cultures: after centrifugation (10 min at 7,000 *g*), the pellets were washed twice with sterile saline water (SSW, 0.85% NaCl in distilled water) and then resuspended in an appropriate volume of SSW to give the desired concentration [expressed as Colony Forming Units (CFU) per ml]. The concentration of each suspension was verified by plating on KB agar plates 100 μl of SSW serial dilutions and counting single colonies after 2 days of incubation at 26°C.

Bacterial epiphytes naturally occurring on *P. savastanoi *host plants (olive, oleander and ash) were also isolated and included in this study. To this purpose two chemically untreated plants for each species were sampled, randomly removing three leaves per plant from one-year-old twigs. Each leaf was then resuspended in SSW (50 ml in a 100 cc Erlenmeyer flask) and incubated at 26°C on a rotatory shaker (200 rpm) for 18 hours. The leaves washings were then separately centrifuged (8,000 *g*, 15 min), each pellet resuspended in 200 μl of SSW, and then used for plating on KB agar, containing cycloheximide (50 μg/ml) to avoid fungal growth. After an incubation of 2 days at 26°C, 50 individual and different bacterial colonies from each leaf washing were randomly isolated and submitted as unidentified pool to DNA extraction.

For long term storage bacteria were maintained at -80°C on 20% (v/v) glycerol.

In order to confirm their previous identification, almost-full-length 16S rRNA genes were amplified from all these isolates and amplifications were performed as described elsewhere [23]. The *P. savastanoi *strains used were also inoculated into 1-year-old olive, oleander and ash stems and tested for their pathogenicity and their virulence, as already described [21].

### DNA extraction from bacteria and plants

Genomic DNA was extracted and purified from 1 ml of bacterial titrated cultures (from 10^6 ^to 10^10 ^CFU/ml), using Puregene^® ^DNA Isolation Kit (Gentra System Inc., MN, USA), according to manufacturers' instructions. DNA concentration was measured both spectrophotometrically, using a NanoDrop™ ND-1000 (NanoDrop Tecnologies Inc., DE, USA) and visually by standard agarose gel electrophoresis [1% agarose (w:v) in TBE 1X] [60].

Bacterial DNA to be used immediately in PCR assays was also obtained by thermal lysis of the pellets from 1 ml of the above mentioned titrated cultures. Each pellet was carefully resuspended in sterile distilled water (100 μl/pellet), incubated at 95°C for 10 min and immediately cooled on ice. After a quick spin in a microcentrifuge, 1 μl lysate was directly used in PCR assays as template.

DNA from *P. savastanoi *host (olive, oleander and ash) and non-host (oak) plants was extracted using Puregene^® ^DNA Isolation Kit (Gentra System Inc.), according to procedure suggested by manufacturers for vegetable materials.

Prior to be used in PCR specific assays, DNA was always checked for its amplificability and the absence of PCR inhibitors, then testing only those giving positive results. Bacterial DNA was amplified using bacterial 16S rDNA universal primers [58] and plant DNA preparations were tested after being spiked with 50 ng of the bacterial DNA target of the primer pair used.

### ERIC-PCR experiments and design of pathovar-specific primers

The Rep-PCR experiments were carried out according to Louws *et al*. (1994) [61], with slight modifications and using Enterobacterial Repetitive Intergenic Consensus (ERIC) primers. The primers ERIC1R and ERIC2 [48] were synthesized by PRIMM (PRIMM srl, Milan, Italy). Amplifications were performed in a programmable thermal cycler Biometra T Professional Basic (Biometra, Goettingen, Germany), in thin-walled 0.5-ml Eppendorf tubes (Sarstedt, Numbrech, Germany), in a 25 μl volume with 50 ng of DNA template per reaction. The reaction mixture and the cycling protocol were already described [62]. Negative controls were included in all PCR amplifications to test for contaminants in the reagents used. For each bacterial isolate, amplification reactions were conducted at least twice, in three separate experiments. Aliquots (10 μl) of PCR products were analysed by electrophoresis in 2% (w:v) agarose gels with 1 × TAE buffer [60], stained with ethidium bromide. The results were visualized, recorded by a video camera and processed by Alphaimager™ system (Alpha Innotech Corporation, San Leandro, CA, USA). The length of the DNA fragments was estimated by comparison with 1 Kb Plus DNA Ladder (Invitrogen Inc, Carlsbad, CA, USA). Amplification profiles were analysed by visual examinations and those amplicons supposed to be pathovar-specific were purified from agarose gel with PureLink^® ^Quick Gel Extraction Kit (Invitrogen) and cloned using TOPO^® ^TA Cloning Kit (Invitrogen) and chemically competent *E. coli *DH5-a cells, under the conditions recommended by the manufacturer. Recombinant plasmids were purified with NucleoSpin Plasmid kit (Macherey-Nagel GmbH, Duren, Germany) and used as template with universal primer T7 and SP6 to generate probes individually labelled with a nonradioactive digoxigenin (DIG) kit (Roche Diagnostics, Mannheim, Germany), according to the manufacturer's instructions. Dot blot analyses were then performed on genomic DNA from *Psv*, *Psn *and *Psf *representative strains blotted on nylon membranes [60]. ERIC-clones generating pathovar-specific probes were then double-strand sequenced at Eurofins MWG Operon Ltd (Ebersberg, Germany). Multiple sequence alignments and comparisons were performed using the computer package CLUSTALW (version 2) [63]http://www.ebi.ac.uk/Tools/clustalw2 and by means of Basic Local Alignment Search Tool (BLAST) http://www.ncbi.nlm.nih.gov/blast analyses to explore all the available DNA sequences in international databases. According to this analysis and using Beacon Designer 7.5 software (Premier Biosoft International, Palo Alto, CA, USA) pathovar-specific primer pairs and probes were designed and synthesized (PRIMM srl), to be used in End Point and Real-Time PCR assays, with SYBR^® ^Green I detection dye and TaqMan^® ^hybridisation probes (Table 2).

### End Point and Real-Time PCR: assay conditions

End Point PCR amplifications were carried out in a 25 μl reaction mixture which contained DNA template (in variable amounts according to the specific experimental purposes), 67 mM TrisHCl, pH 8.8, 16 mM (NH_4_)_2_SO_4_, 0.01% Tween 20, 1.5 mM MgCl_2_, 200 μm of each dNTP, 0.5 μM of each primer, 1 unit Taq DNA polymerase (EuroTaq, Euroclone SpA, Milan, Italy). Amplification was performed in a thermal cycler (Biometra T Professional Basic, Biometra, Goettingen, Germany), using a cycle profile of 95°C (30 sec), 60°C (30 sec) and 72°C (1 min) for 40 cycles, plus an initial step of 95°C for 3 min and a final step of 72°C for 10 min. PCR reaction products (5 μl) were detected by 1.5% agarose gel electrophoresis in TAE 1X stained with ethidium bromide (0.5 μg/ml) and sequenced for confirmation at Eurofins MWG Operon Ltd (Ebersberg, Germany).

Real-Time PCR experiments were performed using the iQ5 Cycler - Real-Time PCR Detection System (Bio-Rad, Hercules, CA, USA), in PCR plates (96 well), with 25 μl reaction mixture volume, the primers and the probes reported in Table 2, and variable DNA amounts depending on the experimental purposes. Each sample, including standards and those DNA-free used as negative control, were run in triplicate and assayed in three independent experiments.

SYBR^® ^Green Real-time PCR was performed using iQ SYBR^® ^Green Supermix (Bio-Rad) according to the manufacturer's instructions. TaqMan^® ^Real-time PCR was performed using iQ^® ^Multiplex Powermix (Bio-Rad), under the conditions recommended by the manufacturer.

### End Point and Real-Time PCR: specificity and detection limits

The specificity of the PCR assays here developed was tested on genomic DNA from *P. savastanoi *strains listed in Table 1, on genomic DNA from olive, oleander, ash and oak, and on total DNA from pools of unidentified bacterial epiphytes isolated from *P. savastanoi *host plants as already described.

The detection limits of these PCR procedures were evaluated by using as template total genomic DNA of representative strains (*Psv *ITM317, *Psn *ITM519 and *Psf *NCPPB1464), serially tenfold diluted (from 50 ng to 0.05 pg or to 5 fg per reaction) or extracted by thermal lysis from 1 ml titrated bacterial cultures (from 10^6 ^to 10^10 ^CFU/ml, with 1 μl DNA per reaction), according to the experimental purposes.

In Real-Time PCR the threshold cycle (Ct) value of each sample depends on the initial amount of the target sequence in the reaction so that it is inversely proportional to the decimal logarithm (log) of the copy number. According to the Ct values obtained, for each *P. savastanoi *pathovar a standard curve was constructed to calculate the correlation between the amount of bacterial DNA and the Ct value, in order to quantify *P. savastanoi *DNA present in unknown samples by interpolation with the linear regression curve.

### Multiplex Real-Time PCR on artificially inoculated plants

Mature leaves were randomly removed from one-year-old twigs of two chemically untreated olive plants, washed in running tap water for 30 min and rinsed three times in an appropriate volume of SSW. After being air dried on a paper towel and in a laminar air flow cabinet, the leaves were aseptically transferred in Petri dishes (90 mm diameter) containing a sterile filter paper disk (3 leaves/plate). Leaves were then separately inoculated with bacterial suspensions of strain *Psv *ITM317 alone or mixed with strains *Psn *ITM519 and *Psf *NCPPB1464, and incubated for 24 hours at 26°C.

Each leaf was inoculated with 100 μl of bacterial suspension with about 10^8 ^CFU/ml/strain. Negative controls were provided by leaves inoculated with sterile water or uninoculated. Three replicates for each inoculation treatment and three independent trials were performed. Each leaf was resuspended in 10 ml of SSW, incubated at 26°C on a rotatory shaker (200 rpm) for 1 hour. The leaves washings were then separately centrifuged (8,000 *g*, 15 min), each pellet resuspended in 100 μl sterile distilled water and subjected to DNA thermal extraction. One μl of lysate was directly used as template in Multiplex Real-Time PCR experiments, using the three TaqMan^® ^probes developed in this study and according to the protocol described above. As positive controls, genomic DNAs of strains *Psv *ITM317, *Psn *ITM519 and *Psf *NCPPB1464 were used (50 ng/reaction).

## Authors' contributions

ST coordinated the study, participated in the concept development and in the assays design, the analysis and interpretation of the results, and drafted the manuscript. MC participated in the concept development and in the assays design, carried out sample preparation and optimization of PCR experimental procedures, the analysis and interpretation of the results, and helped with the manuscript preparation. IML carried out sample preparation and PCR experimental procedures, and helped with analysis and interpretation of the results. ES was involved in the initial study design, participated in sample selection and performed some of the preliminary experiments. All authors read and approved the final manuscript.
